# Protein Tyrosine Nitration and Thiol Oxidation by Peroxynitrite—Strategies to Prevent These Oxidative Modifications

**DOI:** 10.3390/ijms14047542

**Published:** 2013-04-08

**Authors:** Andreas Daiber, Steffen Daub, Markus Bachschmid, Stefan Schildknecht, Matthias Oelze, Sebastian Steven, Patrick Schmidt, Alexandra Megner, Masayuki Wada, Tadashi Tanabe, Thomas Münzel, Serge Bottari, Volker Ullrich

**Affiliations:** 1 2nd Medical Clinic, Molecular Cardiology, Medical Center of the Johannes Gutenberg University, Mainz 55131, Germany; E-Mails: daub.steffen@googlemail.com (S.D.); matzeoelze@aol.com (M.O.); sebastiansteven@gmx.de (S.S.); tmuenzel@uni-mainz.de (T.M.); 2 Vascular Biology Section, Whitaker Cardiovascular Institute, Boston University Medical Center, Boston, MA 02118, USA; E-Mail: markus.bachschmid@t-online.de; 3 Department of Biology, University of Konstanz, Konstanz 78457, Germany; E-Mails: stefan.schildknecht@uni-konstanz.de (S.S.); Patrick.Schmidt@gmx.de (P.S.); volker.ullrich@uni-konstanz.de (V.U.); 4 Department of Pharmacology, National Cardiovascular Center Research Institute, Suita, Osaka 565-8565, Japan; E-Mail: wada@jsc.ri.ncvc.go.jp; 5 Laboratory of Fundamental and Applied, Bioenergetics, INSERM U1055, Grenoble Universités and Pôle de Biologie, CHU, Grenoble 38400, France; E-Mail: serge.bottari@ujf-grenoble.fr

**Keywords:** nitric oxide, superoxide, peroxynitrite, protein tyrosine nitration, thiol oxidation, peroxynitrite scavengers, prostacyclin synthase

## Abstract

The reaction product of nitric oxide and superoxide, peroxynitrite, is a potent biological oxidant. The most important oxidative protein modifications described for peroxynitrite are cysteine-thiol oxidation and tyrosine nitration. We have previously demonstrated that intrinsic heme-thiolate (P450)-dependent enzymatic catalysis increases the nitration of tyrosine 430 in prostacyclin synthase and results in loss of activity which contributes to endothelial dysfunction. We here report the sensitive peroxynitrite-dependent nitration of an over-expressed and partially purified human prostacyclin synthase (3.3 μM) with an EC_50_ value of 5 μM. Microsomal thiols in these preparations effectively compete for peroxynitrite and block the nitration of other proteins up to 50 μM peroxynitrite. Purified, recombinant PGIS showed a half-maximal nitration by 10 μM 3-morpholino sydnonimine (Sin-1) which increased in the presence of bicarbonate, and was only marginally induced by freely diffusing NO_2_-radicals generated by a peroxidase/nitrite/hydrogen peroxide system. Based on these observations, we would like to emphasize that prostacyclin synthase is among the most efficiently and sensitively nitrated proteins investigated by us so far. In the second part of the study, we identified two classes of peroxynitrite scavengers, blocking either peroxynitrite anion-mediated thiol oxidations or phenol/tyrosine nitrations by free radical mechanisms. Dithiopurines and dithiopyrimidines were highly effective in inhibiting both reaction types which could make this class of compounds interesting therapeutic tools. In the present work, we highlighted the impact of experimental conditions on the outcome of peroxynitrite-mediated nitrations. The limitations identified in this work need to be considered in the assessment of experimental data involving peroxynitrite.

## 1. Introduction

Nitric oxide (•NO, EDRF), a potent vasodilator, is formed from arginine by NO synthases [1]. Under physiological conditions, endothelial NO synthase (NOS3) generates low steady-state •NO levels, dilating the smooth muscle of the vasculature via activation of the soluble guanylyl cyclase [2]. Superoxide (O_2_•^−^), a toxic reactive oxygen species that easily reacts with metal-sulfur-clusters and causes oxidative damage via Fenton chemistry, is formed by NADPH oxidases, xanthine oxidase, uncoupled NOS3 and mitochondria [3,4]. Under physiological conditions, O_2_•^−^ steady state levels originating from these sources are efficiently kept low by the enzymatic and non-enzymatic antioxidant system of the cell. In aging or diseases such as inflammation, hypertension, and atherosclerosis, O_2_•^−^ formation increases dramatically and at the same time inducible NO synthase (NOS2) generates high concentrations of •NO. Both radicals react in a diffusion-controlled fashion to yield the toxic and highly reactive nitrogen species peroxynitrite (ONOO^−^) [5], which has been shown to oxidize various biomolecules including proteins, DNA, lipids, as well as low molecular weight antioxidants [6]. The most prominent protein modifications mediated by peroxynitrite are the nitration and dimerization of tyrosine residues, the oxidation of cysteine thiol-groups, as well as the oxidation of methionine sulfur-groups [7,8]. Moreover, disruption of metal-sulfur-clusters has been demonstrated to result in oxidative inactivation of enzymatic catalysis. These oxidative modifications by ONOO^−^ may modulate or inhibit enzymatic activity [9]. However, since ONOO^−^ has been shown to contribute to pathophysiological conditions in various cardiovascular, neurodegenerative and inflammatory diseases [10–13], the majority of ONOO^−^-mediated oxidations obviously must have functional consequences that result in interference with cellular redox-signaling, cell damage, or in death of the entire organism [14]. In the following sections we will have a closer look at the protein tyrosine nitration.

Diverging reports on protein tyrosine nitration exist, with respect to specificity of detection methods, mechanisms of nitrotyrosine formation and pathophysiological or physiological significance [15–17], in particular in diseases related to oxidative stress [10,18–21]. For example, the nitration and modulation of activity of ERK1/2 and Akt play an important role in angiotensin-II triggered vascular complications [22,23]. Commonly used antibodies for semi-quantitative detection of 3-nitrotyrosine in tissues offer a great sensitivity but exert also an epitope preference, which leads to underestimation and misinterpretation of protein nitration. In contrast, total hydrolysis and subsequent HPLC-analysis offers a method for quantification of 3-nitrotyrosine [24,25]. Pitfalls in hydrolysis include losses of 3-nitrotyrosine by partially reducing conditions and false positive results due to the presence of nitrite and its nitrosating/nitrating properties under acidic conditions. Therefore, pronase digestion has been recommended [25,26].

The mechanism of Tyr-nitration was first found to proceed via peroxynitrite on a free radical based mechanism [24,27] but it turned out that heme-containing proteins facilitated this reaction by the formation of ferryl intermediates [25,28]. Prostacyclin synthase (PGIS) was proven sensitive to nitration by peroxynitrite which could be efficiently prevented by an inhibitory substrate analogue [29]. This suggested that tyrosine-nitration was a metal catalyzed process in close proximity to the active heme-iron site [29,30].

Considering the importance of prostacyclin as a vasodilator in the vasculature and heart [31], this oxidative inhibition of the enzyme suggested that it might be of high importance for physiology and pathophysiology. Using other P450 proteins as models, P450_NOR_, P450_BM-3_ and P450_CAM_, we were able to identify compound II (FeO_IV_) of the heme moiety as an essential intermediate of the catalysis of intrinsic tyrosine nitration or peroxynitrite degradation by P450 enzymes [32–34]. Although metal-catalyzed tyrosine nitration represents a major pathway of peroxynitrite-mediated nitration of specific protein tyrosine residues [9,33,34], it should be noted that also carbon dioxide catalysis (with nitroso-peroxocarbonate as an intermediate) [35–37] and conversion of tyrosine to the more reactive tyrosinate residues in the neighborhood of Asp and Glu residues [38] play an important role for the physiological occurrence of this oxidative protein modification [9]. It is also important to note that not all metal-containing enzymes are nitrated in a similar autocatalytic fashion as observed for PGIS, P450_BM-3_ and P450_CAM_. For example P450_NOR_ and chloroperoxidase as well as hemoglobin rather catalyze the decomposition of peroxynitrite without significant nitration of the protein [32,33,39,40].

Concerning the biological significance, PGIS activity was attenuated after nitration of Tyr430 by peroxynitrite [41] which contributed to endothelial dysfunction [42]. Another prominent target of peroxynitrite-dependent inhibition is manganese superoxide dismutase (MnSOD), the mitochondrial isoform of SOD. Although inhibition of this enzyme was not only associated with Tyr34 nitration, but could also involve dityrosine formation [43,44], it has been demonstrated that reaction with peroxynitrite is catalyzed by the manganese cation [45].

One of the major caveats of assigning biological responses to tyrosine nitration, is the occurrence of other peroxynitrite-mediated posttranslational modifications which include thiol oxidation [46–48], sulfoxidations of methionine [49,50] and dityrosine formation [51,52], which can also lead to enzyme inhibition. Moreover, protein Tyr nitration might also leave the enzyme activity entirely unaffected. Therefore, various protein nitrations observed in models of endotoxemia or inflammation may only partially be connected to pathophysiological alterations [53,54]. Such conditions have been referred to as “nitrosative” stress, although different conditions for either nitration and nitrosation were required [26,48] and might hence reflect diverse pathological states and situations. Nevertheless, nitration and nitrosation are both dependent on enhanced production of •NO by NOS1 or NOS2. Regarding the nitration of endothelial PGIS, this can even involve only NOS3 and hence its mechanism of nitration rather reflects a mechanism of redox regulation than a consequence of oxidative stress [55].

With respect to the aforementioned literature, it could be of clinical importance to develop highly specific peroxynitrite scavengers in order to block peroxynitrite-mediated nitration and thiol oxidation. According to these chemical reactions—nitration proceeds via one-electron steps whereas thiol oxidation may involve one- as well as two-electron oxidation steps—the chemical properties of the scavenger groups will probably differ largely. A prominent member of one-electron scavengers is uric acid which reacts with peroxynitrite-derived free radicals [56,57] whereas ebselen is probably the most potent synthetic scavenger of peroxynitrite anion and hence inhibitor of two-electron oxidations [58,59].

In the present study we revisited PGIS and P450_BM-3_ as models of heme-thiolate catalyzed tyrosine nitration, with special emphasis on how these nitrations might be inhibited. In addition, we also investigated the inhibition of nitration and thiol oxidation reactions by various compounds using models for these oxidative modifications: inactivation of alcohol dehydrogenase by thiol oxidation at the active site, as well as peroxynitrite-mediated nitration of phenol or bovine serum albumin.

## 2. Results

### 2.1. Tyrosine Nitration of PGIS

**Figure 1:**
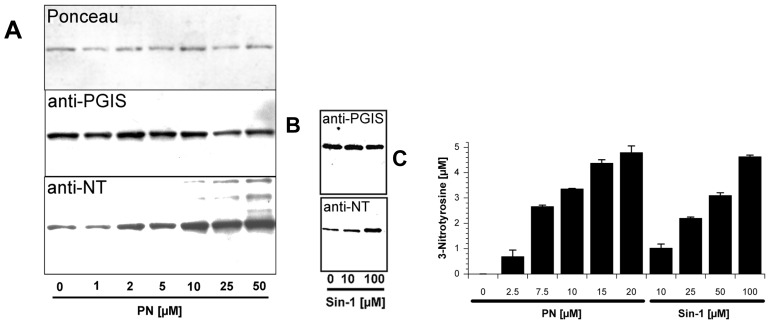
Detection and quantification of tyrosine nitration in human prostacyclin synthase (PGIS). (**A**) Western blot analysis of microsomal fractions (1 mg/mL total protein) containing 200 μg/mg PGIS which were treated with increasing amounts of authentic peroxynitrite (PN, 0–50 μM) or (**B**) Sin-1 (0–100 μM). The figures show staining with Ponceau S, with a polyclonal anti-PGIS antibody and with a monoclonal anti-3-nitrotyrosine antibody; (**C**) HPLC-based quantification of 3-nitrotyrosine in microsomal fractions (1 mg/mL) containing 200 μg/mg PGIS which were treated with increasing amounts of authentic peroxynitrite (PN, 0–20 μM) or peroxynitrite generated *in situ* by Sin-1 (10–100 μM). Data are means ± SEM (**C**) or representative of three independent experiments (**A** and **B**).

Human PGIS was overexpressed in *Spodoptera frugiperda* 21 (Sf21) cells and enriched microsomal fractions, offering a sufficient degree of purity, were used to study peroxynitrite mediated PGIS nitration Bolus added peroxynitrite and Sin-1 [60] caused a concentration-dependent nitration of PGIS, starting at concentrations as low as 2.5 and 10 μM, respectively (Figures 1 and S1). Even in untreated samples, a slight staining was observed due to nitration artifacts during the preparation of microsomes. Protein loading was controlled by Ponceau S staining and by a polyclonal PGIS-antibody (Figure 1A). Slight differences in sample loading were observed and paralleled to small variations in detected nitrotyrosine. Quantitative HPLC analysis of 3-nitrotyrosine from pronase digested microsomal PGIS perfectly matched up with the Western blot. Bolus treatment with peroxynitrite revealed that a 3-fold molar excess of peroxynitrite already caused nitration of a single tyrosine per PGIS molecule (10 μM, see Figure 1C). Saturation of nitration was observed at 50–100 μM peroxynitrite and corresponded to two nitrotyrosine molecules per protein (not shown).

**Figure 2:**
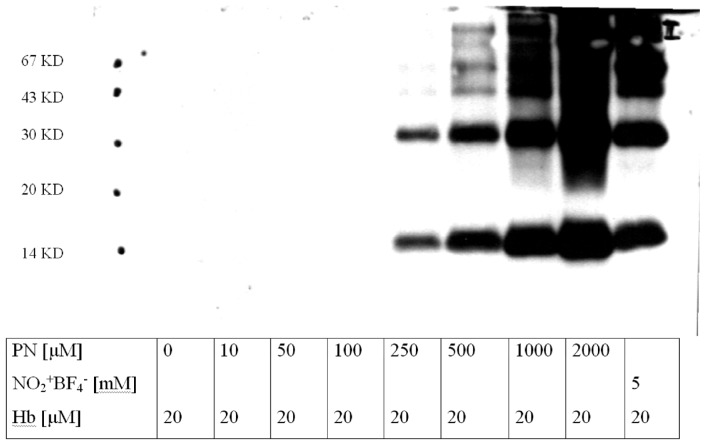
Detection and quantification of tyrosine nitration in bovine hemoglobin (Hb). Western blot analysis of purified Hb (20 μM) treated with increasing amounts of authentic peroxynitrite (PN, 0–2000 μM) or nitronium tetrafluoroborate (5 mM). The figure shows the hybridization with a monoclonal anti-3-nitrotyrosine antibody. Stained bands correspond to the α- and/or β-subunits at 16 kDa as well as to their dimers at around 30 kDa. For corresponding pronase digestion data see Figure S2 in supplementary information.

Higher concentrations of Sin-1 (50 μM roughly equals a steady state release of peroxynitrite—in the range of 50 nM/s; Figure 1C) were required to achieve full nitration of a single Tyr/PGIS molecule and saturation occurred at 500 μM and corresponded to 1.8 mol of 3-nitrotyrosine per mol of PGIS (not shown). Noteworthy is that nitration of purified bovine hemoglobin required at least 250 μM peroxynitrite to become detectable by Western blot (Figure 2).

These results confirm the high reactivity of PGIS towards peroxynitrite due to heme-thiolate catalysis and support our previous findings that the nitrated enzyme is already present in isolated tissue- and cell-fractions. However, this may also indicate some background or cross-reactivity of the nitrotyrosine antibodies used (since HPLC analysis revealed no nitration in the control samples, Figure 1C) and demonstrated the limitations of qualitative detection of nitrated proteins by these antibodies.

**Figure 3:**
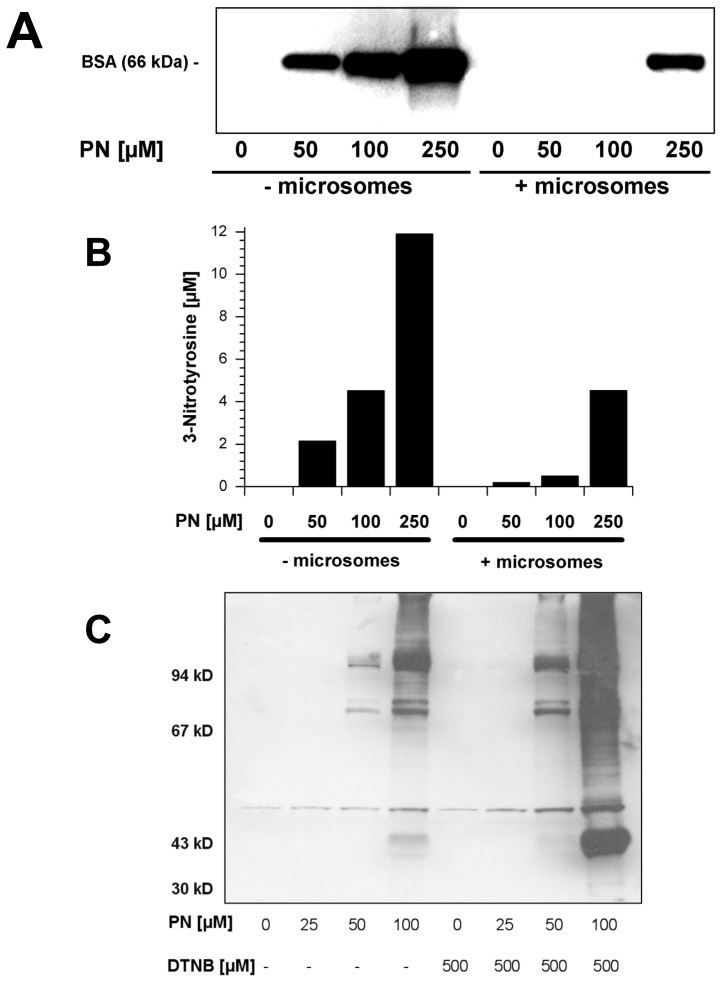
Effects of bovine aortic microsomes on the nitration of bovine serum albumin (BSA) and of Ellman’s reagent on the nitration of PGIS and other proteins by peroxynitrite. (**A**) Detection of 3-nitrotyrosine in BSA by Western blot analysis using a monoclonal anti-3-nitrotyrosine antibody. BSA (5 μM) was incubated with authentic peroxynitrite (PN, 0–250 μM) in the presence or absence of bovine aortic microsomes (1 mg/mL total protein); (**B**) HPLC analysis of free 3-nitrotyrosine content in pronase digests from samples used for Western blot; (**C**) Detection of 3-nitrotyrosine positive proteins in bovine aortic microsomes by Western blot analysis using a monoclonal anti-3-nitrotyrosine antibody. Bovine aortic microsomes (1 mg/mL total protein), which were either treated or not with the sulfhydryl oxidizing Ellman’s reagent (DTNB) were mixed with authentic peroxynitrite (PN, 0–100 μM). Data are representative of two independent experiments.

The sensitivity of microsomal PGIS preparations towards nitration was always significantly lower than that of purified PGIS (reported IC_50_ values ≈500 nM *vs*. ≈50 nM). The microsomal “quenching effect” towards nitration was demonstrated by treating bovine serum albumin (5 μM) either in the absence or presence of bovine coronary artery microsomes with peroxynitrite (Figure 3A). Addition of microsomes required about 5-fold higher concentrations of peroxynitrite to cause a similar degree of nitration than for pure albumin. Since microsomal concentration of endogenous PGIS is negligible compared to the amount of albumin (see Ponceau S staining in Figure S3, supplementary information), nitrated PGIS was below the detection limit of Western blots. These results are in perfect agreement with the results obtained by pronase digestion and quantitative HPLC analysis of nitrotyrosine (Figure 3B).

Peroxynitrite has been shown to efficiently react with sulfhydryl groups. To prove the contribution of free protein thiol groups to the antioxidant potential of microsomal suspensions, sulfhydryls were blocked by Ellman’s reagent (DTNB). In these preparations, peroxynitrite-mediated nitration of PGIS and other proteins was significantly increased, indicating that protein bound thiol groups can efficiently scavenge peroxynitrite (Figure 3C).

**Figure 4:**
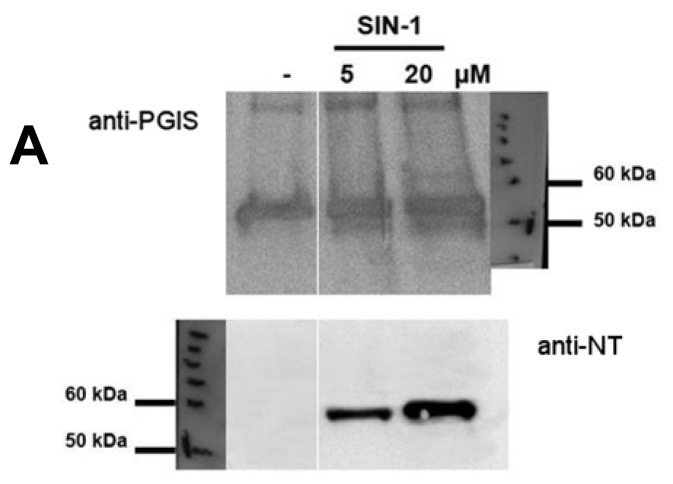

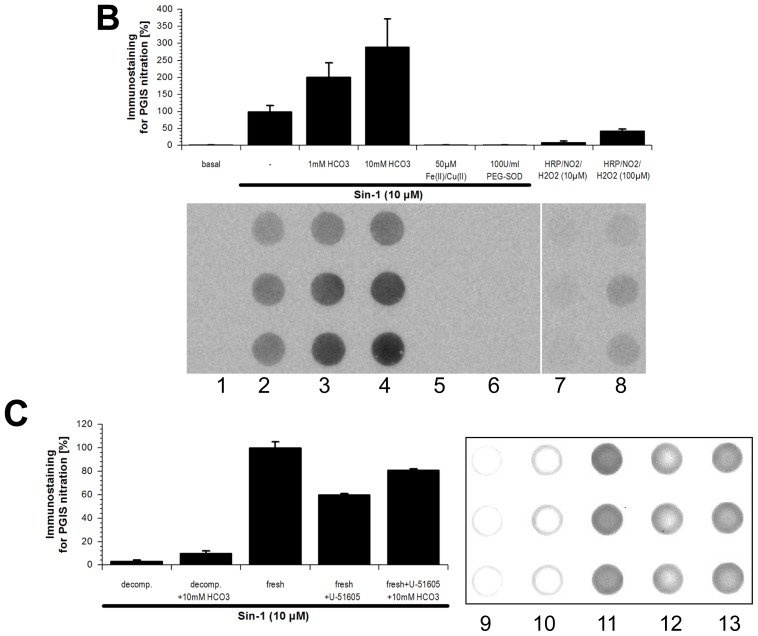
Nitration of purified, recombinant PGIS by *in situ* generated peroxynitrite. (**A**) Detection of 3-nitrotyrosine in PGIS by Western blot analysis using a polyclonal amti-PGIS antibody and a monoclonal anti-3-nitrotyrosine antibody. PGIS (300 nM) was treated with peroxynitrite generated *in situ* from Sin-1 (5 and 20 μM). Data are representative of two independent experiments; (**B**) Detection of 3-nitrotyrosine in PGIS by dot blot analysis using a monoclonal anti-3-nitrotyrosine antibody. PGIS (80 nM) was not treated (lane 1) or treated with peroxynitrite generated *in situ* from Sin-1 (10 μM) (lane 2) in the absence or presence of 1 mM (lane 3) or 10 mM (lane 4) bicarbonate or 50 μM iron(II) plus copper (II) ions (lane 5) or 100 U/mL PEG-SOD (lane 6). PGIS was also incubated with 0.1 μM horseradish peroxidase (HRP) plus 10 μM nitrite/hydrogen peroxide (lane 7) or plus 100 μM nitrite/hydrogen peroxide (lane 8); (**C**) Detection of 3-nitrotyrosine in PGIS by dot blot analysis using a monoclonal anti-3-nitrotyrosine antibody. PGIS (80 nM) was treated with 10 μM decomposed Sin-1, from a 1 mM Sin-1 solution in 1 M potassium phosphate buffer pH 7.4 incubated for 90 min at 37 °C, in the absence (lane 9) or presence of 10 mM bicarbonate (lane 10), freshly prepared 10 μM Sin-1 in the absence (lane 11) or presence of 2 μM U-51605 (lane 12) as well as 2 μM U-51605 plus 10 mM bicarbonate (lane 13). All incubations were performed in 0.1 M potassium phosphate buffer pH 7.4 at 37 °C for 90 min. Data are means ± SEM of three independent experiments.

Using purified recombinant PGIS, we were able to determine half-maximal nitration by peroxynitrite generated *in situ* from Sin-1 (10 μM, Figure 4A) which was increased by bicarbonate in a concentration-dependent fashion, indicating a role of carbon dioxide for the nitration process of PGIS (Figure 4B). In contrast, free iron(II)/copper(II) ions and polyethylene glycolated Cu,Zn-superoxide dismutase (PEG-SOD) completely abolished the nitration of PGIS. Peroxidase-catalyzed nitrogen dioxide radical formation (even at higher concentrations of nitrite and hydrogen peroxide) only caused marginal nitration levels of the purified enzyme (Figure 4B). Decomposed Sin-1 yielded only background 3-nitrostyrosine staining, which showed no appreciable increase in the presence of bicarbonate (Figure 4C). In contrast, PGIS nitration was increased by fresh Sin-1 (10 μM) and was blocked by almost 40% in the presence of the active site inhibitor of PGIS, U-51605. Bicarbonate caused a minor increase in the nitration signal, indicating that the nitrosoperoxycarbonate (ONOOCO_2_^−^) species also required access to the heme-thiolate active site of PGIS in order to mediate efficient nitration of the enzyme (Figure 4C).

Regarding the biological importance of PGIS nitration and activity as well as the known nitration site at Tyr430, future effort should be focused on the identification of a specific marker, such as a nitrated peptide, of inactivated PGIS. As reported before [41], thermolysine digestion yielded the unusual fragment NH_2_-LKNY(NO_2_)-OH and could be detected by MS at 291.6 *m*/*z*, indicating that the nitro group introduced an additional thermolysine cleavage site (supplementary Figure S4). Detailed information on stability and implications of nitrated PGIS peptide is provided in supplementary information (supplemental Section 1.2).

### 2.2. Nitration of P450_BM-3_ by *in Situ* Generated Peroxynitrite from Sin-1 or Xanthine Oxidase/Spermine NONOate

**Figure 5:**
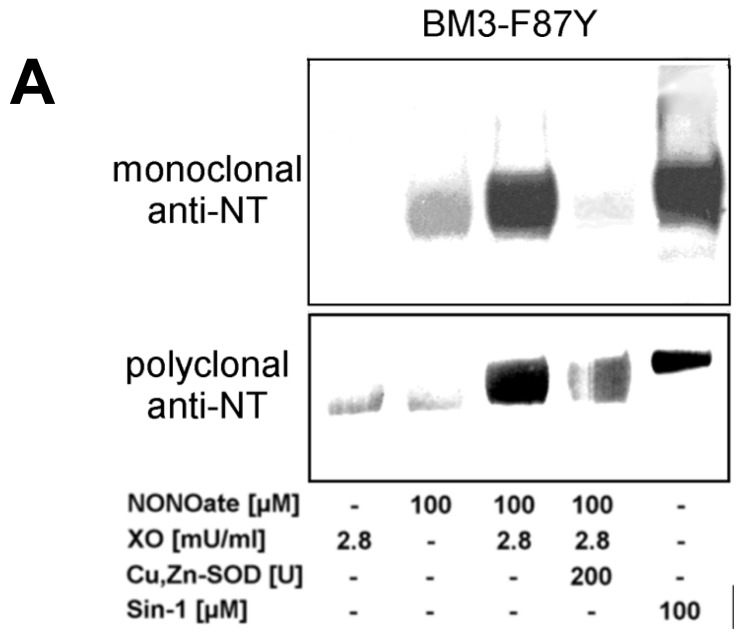

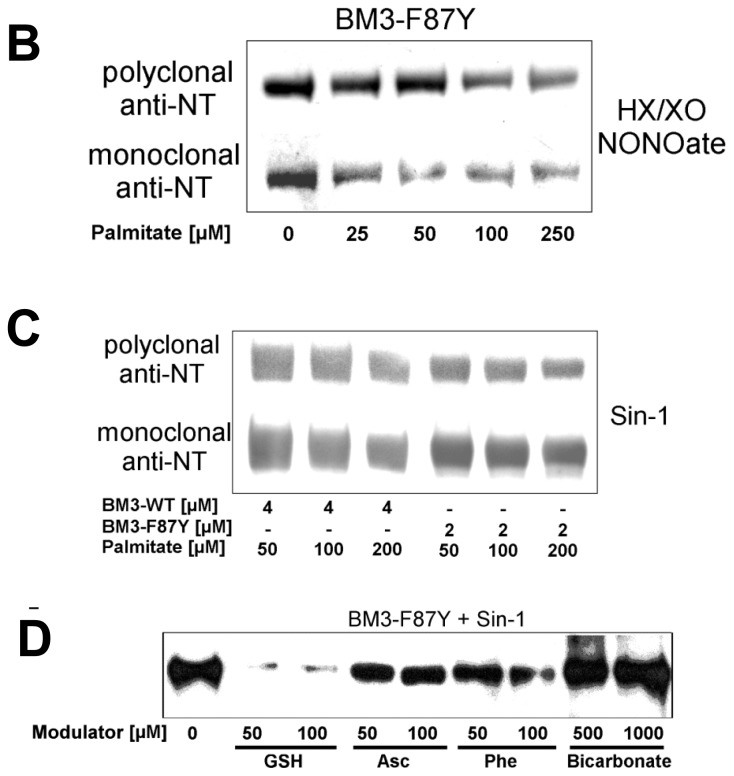
Effect of palmitate on the nitration of purified wildtype and F87Y variant P450_BM-3_ by *in situ* generated peroxynitrite. (**A**) Detection of 3-nitrotyrosine in P450_BM-3_ F87Y variant by Western blot analysis using monoclonal and polyclonal anti-3-nitrotyrosine antibodies. P450_BM-3_ F87Y variant (2 μM) was treated with peroxynitrite generated *in situ* using xanthine oxidase (XO, 2.8 mU/mL) and spermine NONOate (100 μM) in the presence or absence of Cu,Zn-SOD and (**B**) in the presence of increasing amounts of palmitate (0–250 μM); (**C**) Detection of 3-nitrotyrosine in P450_BM-3_ by Western blot analysis using monoclonal and polyclonal anti-3-nitrotyrosine antibodies. P450_BM-3_ was treated with peroxynitrite generated *in situ* using Sin-1 (100 μM) in the presence of increasing palmitate concentrations (50–200 μM); (**D**) Effect of antioxidants and bicarbonate on the nitration of P450_BM-3_ F87Y variant by *in situ* generated peroxynitrite. Detection of 3-nitrotyrosine in P450_BM-3_ F87Y variant by Western blot analysis using a monoclonal anti-3-nitrotyrosine antibody. P450_BM-3_ F87Y variant (0.5 μM) was treated with peroxynitrite generated *in situ* using Sin-1 (100 μM) in the absence or presence of glutathione (GSH), ascorbate (Asc), phenol (Phe) or bicarbonate. HX means hypoxanthine. Data are representative of two independent experiments.

Nitration of P450_BM-3_ enzymes (wild type and F87Y mutant) and the molecular mechanisms involved were previously investigated [26,33]. The P450_BM-3_ F87Y mutant has a higher sensitivity towards nitration since the introduced tyrosine is located close to the active site. Both *in situ* nitration systems (XO/NONOate or Sin-1) efficiently nitrated the enzyme whereas only a minor effect was observed when the enzyme was incubated with the NO-donor alone (Figure 5A). Superoxide dismutase nearly entirely blocked XO/NONOate and Sin-1-induced nitration (Figure 5A). Detailed information is provided in the supplementary information (supplemental section 1.1 and Figures S5 and S6). Nitration was inhibited by co-incubation of the enzyme with its endogenous substrate palmitate, which reduces the accessibility to the active site to peroxynitrite (Figure 5B,C). These results are in agreement with the previous finding that tyrosine nitration depends on heme-thiolate catalysis. Furthermore, the nitration of P450_BM-3_ F87Y mutant by Sin-1 could be efficiently prevented by addition of glutathione and with a lower efficacy by phenol, or ascorbate whereas bicarbonate significantly increased nitration (Figure 5D). Nitration was most efficiently suppressed by a system consisting of horseradish peroxidase and phenol, glutathione or ascorbate (not shown). This system was described as a highly efficient sink for peroxynitrite [61] although it might also be pro-oxidative [62]. Previous studies [26] and additional Western blot analysis demonstrated that bolus additions of peroxynitrite (500 μM) nitrated various proteins indistinctively with only minor differences in nitration sensitivity (Figure S7 in supplementary information). In contrast, peroxynitrite generated *in situ* from Sin-1 (250 or 500 μM) led to selective nitration of only certain proteins, clearly indicating the dependence on metal-catalysis in conjunction with vicinal tyrosine residues (Figure S7 in supplementary information).

### 2.3. Scavengers of Peroxynitrite Inhibiting the Nitration and Hydroxylation of Phenol

Phenol was used instead of tyrosine since its solubility is superior in aqueous solutions and the availability of the para-position (with regard to the hydroxyl group) allowed us to study the nitrosative reactivity of peroxynitrite through the formation of the stable 4-nitrosophenol product (3-nitrosotyrosine is unstable). Moreover, the standards of reaction products are commercially available and the reaction mechanism of phenol with peroxynitrite is even better characterized than that of tyrosine. Finally, phenol nitration has proven to be a valid model for protein tyrosine nitration. Peroxynitrite-mediated phenol nitration has been reported to involve phenoxy radicals [63] and can be considered as two subsequent single-electron oxidation steps conducted by peroxynitrous acid (ONOOH), or more precisely the caged radical of decaying peroxynitrite [24,52,64,65]. The inhibition of nitrophenol formation at pH 6.0 in the peroxynitrite/phenol system was assessed in the presence of several peroxynitrite-scavengers at various concentrations. Two major categories of inhibitors emerged: the first one showed an exponential concentration-dependency (uric acid derivatives, dithio-purine and -pyrimidine), whereas the second one followed a more linear concentration-dependency (thiols, ebselen, methionine, tyrosine and ascorbate) (Figure S8 in supplementary information). This behavior is directly related to their reactivity towards ONOOH-derived free radicals or ONOO^−^. Table 1 shows the IC_50_-values of some natural antioxidants and synthetic counterparts for phenol nitration by peroxynitrite. It turned out, that among the best inhibitors of phenol nitration are uric acid and its 1,3- and 3,7-dimethyl analogues (DMUA), whereas Se-methionine and methionine itself showed low scavenging efficacy in this system. Interestingly, xanthine, allopurinol, caffeine, allantoin and alloxan, which are all structurally related to uric acid, were without any effect in accordance with a previous report [66].

In a different set of experiments, constant concentrations of peroxynitrite, phenol and scavengers were used to measure the hydroxylation of phenol by peroxynitrite (Figure S9 in supplementary information). Again, uric acid was the most effective in suppressing hydroxy-products and benzoquinone. Ascorbate, GSH, cysteine, ebselen and methionine showed only minor effects on hydroxylation. In the case of 2,6-DTPu and -DTPy, hydroxy-products and benzoquinone could not be detected by HPLC due to interfering product peaks. Additional experiments demonstrated no significant effects on peroxynitrite-mediated hydroxylation or nitration by phosphoenolpyruvate, oxalate, acetone or α-ketoglutarate, but a small decrease in nitration by acetaldehyde and a trialkyl-phosphine (not shown). Pyruvate showed a minor increase in nitration (not shown). Very low IC_50_-values for TEMPO in the system of phenol nitration have been reported [67] and were confirmed in our system (IC_50_-values of less than 2 μM). Nevertheless, 4-nitrosophenol levels where increased, supporting previously postulated mechanism for this reaction [67].

### 2.4. Scavengers of Peroxynitrite Inhibiting the Nitrosation of Phenol

The nitrosation of phenol by peroxynitrite was investigated at pH 9 using the same set of scavengers (Table 1). Again, uric acid derivatives and the thio-purines and -pyrimidines were best in inhibiting phenol nitrosation, suggesting that this process also involves radical species. Furthermore, at alkaline pH, high amounts of biphenols were formed, implicating the involvement of phenoxy radicals [63], for which the same group of scavengers is most effective.

### 2.5. Scavengers of Peroxynitrite Inhibiting the Nitration of BSA

The results on phenol nitration could be reproduced in another test system where the same scavengers attenuated peroxynitrite-mediated BSA nitration (Table 2). Again, uric acid, 1,3-DMUA and 2-TBA were the most efficient compounds in inhibiting the nitration of tyrosine residues in BSA.

### 2.6. Scavengers of Peroxynitrite Inhibiting the Metal-Catalyzed Nitration of Phenol

In an additional series of experiments, a system containing phenol and MP-11 was used as a model for the metal-catalyzed tyrosine nitration of proteins. Nitration of 5 mM phenol by 655 μM peroxynitrite at pH 6 was increased by a factor of 4 to 5 by 5 μM MP-11. The same scavengers as the ones tested above were used in this system and for the first time it turned out that also for the metal-catalyzed nitration, uric acid, 1.3-DMUA, 2.6-DTPu and -DTPy were much more effective in inhibiting phenol nitration than GSH, ascorbate, ebselen, methionine and Se-methionine (Figure S10 in supplementary information). Only 2-TBA showed a surprisingly low efficacy in this system.

### 2.7. Scavengers of Peroxynitrite Inhibiting the Oxidation and Inactivation of Alcohol Dehydrogenase

As a second test-system we used ONOO^−^-mediated oxidation of alcohol dehydrogenase (ADH). The protecting effect of the various scavengers on ADH-activity directly correlated with the reactivity of the scavengers towards the peroxynitrite-anion. Beckman *et al*. previously reported sensitive thiol-oxidation and ADH-inactivation at low peroxynitrite concentrations [68]. We confirmed these findings in a subsequent publication [48] and with the data presented here (Figures S11 and S12 in supplementary information). The IC_50_-value for peroxynitrite was around 1 to 2 μM, for •NO alone (from the hydrolysis of diethyl NONOate). In air-saturated solution it was at least 5-fold higher and in deoxygenated solutions it increased by a factor 10 [48]. Table 3 shows the effect of peroxynitrite scavengers in this system and it turned out that uric acid derivatives, tyrosine and tryptophan showed very low protection of ADH from oxidation by 20 μM peroxynitrite, whereas GSH, cysteine, Se-methionine, and to a lesser extent methionine, were highly efficient in preventing ADH inactivation. As a control we verified that the scavengers used could not reactivate already inactivated ADH. 2.6-DTPy and -DTPu also showed a protective activity in this system, but led themselves to the inhibition of the enzyme, when used at higher concentrations, thereby preventing them from completely preserving ADH activity. Ebselen, which is known to react with thiols to form adducts [59] inhibited ADH already at nM concentrations (Figure S11 in supplementary information). Ascorbate seemed to form intermediates with peroxynitrite (probably ascorbyl radicals), which also inhibited ADH. Therefore, the maximal protection of ADH activity obtained with ascorbate did not exceed 60%.

## 3. Discussion

Nitric oxide (•NO) and superoxide (•O_2_^−^) are two endogenously formed radical species in biological systems. Although they exert minor reactivity towards organic molecules when present alone, both radicals can combine in an almost diffusion-limited process to form peroxynitrite [69]. For this product, a high reactivity towards biological macromolecules has been observed, e.g., DNA strand breaks [70], fatty acids oxidation [71–74], and numerous modifications of proteins such as thiol oxidation [47,48,68], sulfoxidation of methionine residues [7,75,76] and tyrosine nitration [26,29,43,77]. Therefore, peroxynitrite might account for numerous deleterious effects associated with nitro-oxidative stress. Indeed, by using tyrosine nitration as a footprint of endogenous peroxynitrite formation, an association with Alzheimer’s Disease, hypoxia [78], hypoglycemia [79], shock, allograft rejection, stroke and other cardiovascular and neurodegenerative diseases has been postulated (for reviews see [10,11,18,20,24]).

Our interest in mechanistic and analytical details of tyrosine nitration was derived from the high sensitivity and selectivity of PGIS nitration and inhibition [29,80]. Pure enzyme preparations reacted with IC_50_-values of around 100 nM peroxynitrite [81] and even in whole cells and aortic tissue such low values were observed [29,42,80,82]. This was puzzling considering the many antioxidants and potential other peroxynitrite targets in cellular systems. It might however partially be explained by the localization of PGIS in caveolae of the plasma membrane [83], which allow direct access of peroxynitrite to the enzyme. It also should be mentioned that for peroxynitrite an assessment of IC_50_-values has to take into account its high reactivity which leads to rapid changes of its steady state concentrations in the presence of varying concentrations of competing substrates. This becomes important when comparing inhibitory concentrations of peroxynitrite for pure proteins and protein homogenates which is most conclusively demonstrated by the effect of aortic microsomes on exogenous BSA nitration by peroxynitrite. The sensitive nitration and inactivation of PGIS on one hand, and the increased production of the PGIS substrate PGH_2_ by enhanced cyclooxygenase activity in the presence of higher peroxide concentrations (e.g., hydrogen peroxide, lipid peroxides, peroxynitrite), provides the basis for an efficient redox-regulatory system of the vascular tone [55,84,85]. Based on this increased activity of the COX/PGIS pathway due to an increased peroxide tone, some pathological situations such as dilated cardiomyopathy, or genetically-induced hypertension, are associated with increased prostacyclin levels despite increased reactive oxygen species levels and decreased nitric oxide bioavailability [86,87].

Our second aim was to evaluate the use of anti-nitrotyrosine antibodies, since their use has been questioned in terms of specificity and sensitivity. We could observe a good correlation between Western blot staining and 3-nitrotyrosine levels, detected by HPLC analysis after pronase digestion—but there are cases where 3-nitrotyrosine containing epitopes react better with monoclonal than with polyclonal Ab’s and vice versa. Since the catalytic sites of metallo-enzymes are usually buried, a proper unfolding of the protein is required to obtain sufficient sensitivity. This may require repeated stripping of the membrane e.g., in the case of PGIS in order to ensure proper denaturation of the enzyme. We were able to show that PGIS can be nitrated by rather low concentrations of peroxynitrite and that part of the purified enzyme already exhibits tyrosine nitration. Pronase digestion for up to 6 days is required to achieve a complete hydrolysis of PGIS, whereas other soluble proteins like BSA required only 2–3 h. Meanwhile, the nitrated tyrosine has been identified at position 430 by LC-FTICR-MS, which is close to the heme-thiolate active site in the normal 3-dimensional configuration of active PGIS [41].

With this background, the search for pharmacologically active inhibitors of the presumed deleterious actions of peroxynitrite becomes possible. However, the reactions involving peroxynitrite may not all be deleterious as we have presented evidence that PGIS undergoes tyrosine nitration and inactivation at very low peroxynitrite concentrations, suggesting a physiological regulatory role of this posttranslational modification [29,42,80]. This reaction seems to be auto-catalyzed by the active heme site of the enzyme since substrate analogues which blocked the active site decreased its nitration [29]. By using other P450 proteins (P450_CAM_ and P450_BM-3_) we were able to prove that this autocatalytic nitration by peroxynitrite occurs with other members of this family of proteins [33,34]. Based on these observations, the nitration of PGIS seems to fulfill a regulatory role. In order to prove this hypothesis, we tested for scavengers of this process but were not successful [39] except for ebselen which turned out to compete with PGIS nitration. This however occurred only in the absence of thiols, making it unsuitable for cellular or even *in vivo* inhibition experiments [59]. This has stimulated our interest in a rational design of peroxynitrite scavengers but according to the literature and our own results, this topic proved to be difficult due to the different forms under which peroxynitrite can exert its actions.

Considering that peroxynitrite’s oxidizing properties correlate with its readiness to undergo O–O bond cleavage to give a radical pair of HO• and •NO_2_, its reactivity will depend on the neutralization of its anion by a proton (p*K*_a_ = 6.8) [88] or by formation of an oxo species in the presence of suitable transition metal ions, like Mn^2+^ or Mn^3+^, Cu^2+^ or preferentially Fe^3+^[25]. The latter yields ferryl complexes [formally Fe(IV)=O] in heme when reacting with peroxynitrite. The nitration of PGIS has been explained the same way. In the absence of a metal ion, a proton is required and hence pH values below 6 are required to generate sufficient quantities of peroxynitrous acid. This acid form can be thermally excited to dissociate into a radical pair [HO• •ONO]_cage_[24] by which an oxidative attack on organic compounds can be initiated. Efficient scavengers of this type of reactions preferentially react with the radical cage form of peroxynitrite or derived free radicals and usually show a clear pH-dependence in their reactivity (e.g., uric acid, Figure S10 in supplementary information). Alternatively, if suitable substrates are lacking, isomerization to nitrate can occur or it will react with a second peroxynitrite molecule leading to dioxygen and dinitrogen trioxide (N_2_O_3_) which is easily hydrolyzed to nitrite [89]. Since this reaction is unavoidable at the concentrations usually used, the nitrosating properties of N_2_O_3_ also have to be considered [48,63].

In the peroxynitrite literature, all reactions involving homolytic OO-bond cleavage have been summarized as “1e-oxidations” in contrast to a few reactions which transfer one oxygen atom from peroxynitrite and therefore have been termed “2e-oxidations”. The formation of ebselen-oxide from ebselen [58,59] or the sulfoxide from methionine [49,50] are examples of this type of reaction which can originate from the peroxynitrite anion as evidenced by the pH-independence of such oxidations (e.g., ebselen, Figure S11 in supplemental information). To further complicate the reaction pattern of peroxynitrite, its anion can behave as a nucleophile and form adducts, e.g., with carbonyls. The peroxo compound arising from carbon dioxide is a well-studied example of this reaction [35,36].

Considering the plethora of oxidations by peroxynitrite, it is obvious that no specific inhibitor or scavenger will be identified, but only mechanism-based subtypes can be found. Lots of results have been published previously concerning the peroxynitrite scavenging properties of natural and synthetic compounds [56,90,91]. In addition, lots of different test systems have been used to compare these compounds [92], especially the α_1_-antiproteinase-elastase system which was used to test the effects of peroxynitrite scavengers on methionine oxidation and α_1_-antiproteinase inactivation [56,90]. In the present study, phenol was used as a substrate since previous reports suggested that the “1e-oxidation” pathway leads to a phenoxy-radical to which an •NO_2_ radical can add to form 2- and 4-nitrophenol [63]. By measuring this parameter, which also closely mimics the nitration of tyrosine residues in proteins, we attempted to define a basis for peroxynitrite scavengers.

One of the aims of the present study was to reinvestigate a series of organic compounds as potential inhibitors of peroxynitrite actions with a particular emphasis on biologically occurring substances as potential lead structures. Among them there would be compounds such as ascorbate or tocopherols, but also carbonyl derivatives since carbon dioxide is known to form an addition product with the peroxynitrite anion [35,93]. Our main interest, however, was focused on uric acid which had been described as an exceptionally good scavenger of peroxynitrite [56,57,94]. As a test system we measured the hydroxylation and nitration of phenol [63], which not only allowed to monitor the formation of the radical pair but also served as a model for tyrosine nitration in proteins. The analytics and the mechanisms involved were recently published [63] and have been extended to heme catalysis [32,39]. Another aim of this study was to distinguish between scavengers which mainly react with the protonated form of peroxynitrite and those which show high reactivity for the peroxynitrite-anion. For this reason, we used an enzymatic system based on ADH-activity as a second test system. For ADH, Beckman and coworkers reported a *k*-value of 4 × 10^5^ M^−1^ s^−1^ for the reaction with the peroxynitrite-anion [68]. Therefore, this enzyme seemed to be a perfect test system to monitor the scavenging ability and the protective effects of the antioxidant compounds to be tested. Furthermore decomposition kinetics of peroxynitrite at alkaline pH are supposed to indicate if a compound is able to react directly with peroxynitrite-anion or if it is involved in the reaction of the protonated form or one of the decomposition intermediates.

## 4. Materials and Methods

### 4.1. Materials

Wild type P450_BM-3_ (CYP 102) [95] and its F87Y variant [96] from *Bacillus megaterium* were kindly donated by J.A. Peterson (TX, USA) and expressed and purified as described. P450_CAM_ (E.C. 1.14.14.1) from *Pseudomonas putida* was a kind gift of C. Jung (Max-Delbrück-Centrum, Berlin, Germany) and prepared as published previously [97]. P450_NOR_ (CYP 55AI) from *Fusarium oxysporum* was a kind gift of H. Shoun and N. Takaya (University of Tokyo, Tokyo, Japan) and purified as described previously [98]. Human PGIS (E.C. 5.3.99.4) was overexpressed in *Spodoptera frugiperda* 21 (Sf21) cells and partially purified as recently described [99]. The microsomal concentration of PGIS was determined by the tranylcypromine binding-spectrum to be 3.3 μM (200 μg/mL) at a total protein concentration of 1 mg/mL [99]. PGIS activity was determined using ^14^C-prostaglandin endoperoxide H_2_ (PGH_2_) as previously described [99]. Cu,Zn-SOD (EC 1.15.1.1) from bovine erythrocytes, CPO (EC 1.11.1.10) from *Caldariomyces fumago*, xanthine oxidase (E.C. 1.1.3.22) grade III from buttermilk and BSA were purchased from Sigma (Steinheim, Germany). Pronase from *Streptomyces griseus* (lyophylized powder) was obtained from Roche (Mannheim, Germany) and thermolysine from *Bacillus thermoproteolyticus rokko* (type X) was from Sigma (St. Louis, MO, USA). Sin-1 hydrochloride (3-morpholinosydnonimine HCl) was from Calbiochem-Novabiochem Corporation (La Jolla, CA, USA). Spermine NONOate (*N*-[4-[1-(3-aminopropyl)-2-hydroxy-2- nitrosohydrazino]butyl]-1,3-propanediamine) was purchased from Cayman Chemical Company (Ann Arbor, MI, USA). The protease inhibitor cocktail (general use) was prepared according to the product information from Sigma-Aldrich (Steinheim, Germany). Peroxynitrite was a kind gift of M. Mehl (Department of Inorganic Chemistry, ETH Zürich, Switzerland) and synthesized as described by Kissner and Koppenol [69] from •NO and potassium superoxide and depleted of residual H_2_O_2_ with MnO_2_. The dimethyluric acid derivatives and dithiopurine and -pyrimidine were purchased from Sigma-Aldrich. Ebselen was a kind gift from A. Wendel (University of Konstanz, Konstanz, Germany). TEMPONE and TEMPO were purchased from Alexis (Loerrach, Germany). All other chemicals were of analytical grade.

### 4.2. Nitration of Purified, Recombinant PGIS Protein by *in Situ* Generated Peroxynitrite from Sin-1

Purified, recombinant PGIS protein was a kind gift from Dr. C.S. Raman (Department of Pharmaceutical Sciences, University of Maryland, Baltimore, MD, USA) and Dr. P. Nioche (Université Paris Descartes, INSERM UMR-S 747, Paris, France) and prepared according to previous reports with some modifications [100]. Conditions of incubation were as described in the legend to Figure 4 and Western blot analysis as well as dot blot analysis were performed as described previously [101,102] using a monoclonal anti-3-nitrotyrosine antibody at a dilution of 1:1000 (Upstate Biotechnology/Millipore, Lake Placid, NY, USA) and a polyclonal anti-PGIS antibody at a dilution of 1:250 (Cayman Europe, Tallinn, Estonia).

### 4.3. Nitration of P450_BM-3_ Proteins and PGIS by either Authentic Peroxynitrite or *in Situ* Generated Peroxynitrite from Sin-1 or Xanthine Oxidase/Spermine NONOate

Human PGIS (3.3 μM, 200 μg/mg total protein) overexpressed in *Spodoptera frugiperda* 21 (Sf21) cells was used as a partially purified microsomal suspension (1 mg/mL total protein) in 0.1 M potassium phosphate buffer pH 7.4. Bovine PGIS was used as a suspension of aortic microsomes (1 mg/mL total protein) in 0.1 M potassium phosphate buffer pH 7.5 as previously described [41]. Purified P450_BM-3_ proteins were used at a concentration of 2.5 μM. A small aliquot of peroxynitrite (100 mM in 0.1 M NaOH) was added by rapid mixing of the reaction solutions and was allowed to completely decompose. Sin-1 (100 μM from a 0.1 M acidic stock solution) or xanthine oxidase (2.8 mU/mL) and/or spermine NONOate (100 μM from a 0.1 M alkaline stock solution) were added to the protein solutions and incubated for 90 min at 37 °C to allow complete decomposition.

### 4.4. Inhibition of Nitration by Peroxynitrite Scavengers and Substrate Analogue Inhibitors

Palmitate, a natural substrate of P450_BM-3_, was used from a stock solution in DMSO. Palmitate (25–250 μM) was preincubated with wild type P450_BM-3_ or its F87Y variant (4 and 2 μM, respectively) and all samples were adjusted with pure DMSO, so that all of them contained 1% *v*/*v* of DMSO. These amounts of DMSO and palmitate did not interfere with peroxynitrite-, Sin-1- or xanthine oxidase/spermine NONOate-mediated nitration of P450_BM-3_. Peroxynitrite scavengers (50 and 100 μM) such as ascorbate, glutathione and phenol were added together with P450_BM-3_ F87Y variant (0.5 μM) before mixing with Sin-1 (100 μM). BSA (5 μM) was treated with peroxynitrite (50–250 μM) in the presence and absence of bovine coronary microsomes (1 mg/mL total protein).

### 4.5. Western Blot Analysis of Nitrated Proteins

The nitration of P450_BM-3_ proteins and PGIS was detected by Western blot analysis, using a mouse monoclonal anti-3-nitrotyrosine antibody from Upstate Biotechnology (Hamburg, Germany) at a dilution of 1:1000 together with an HRP-conjugated secondary antibody (GAM-POX, 1:7500) which was obtained from Pierce (Rockford, IL, USA). Additionally a rabbit polyclonal anti-3-nitrotyrosine antibody from Upstate Biotechnology was used at a dilution of 1:1000 together with an HRP-conjugated secondary antibody (GAR-POX, 1:3000) from Pierce (Rockford, IL, USA). PGIS was detected with a rabbit polyclonal antibody which was produced according to the method of Siegle *et al*. and was used at a dilution of 1 μg/mL PBS [103]. Proteins were separated by SDS-PAGE on Novex 8% Tris-glycine gels (12 wells) from Invitrogen Corporation (Carlsbad, NM, USA). 2.5–10 μL of each sample in Laemmli buffer were loaded on the gels, the running buffer consisted of 25 mM Tris, 192 mM glycine and 5 mM SDS at a current of 35 mA for 1 h. Proteins were transferred by the semi-dry blotting procedure to a nitrocellulose membrane as described previously [41]. Finally the blot was developed with Super Signal ECL kit from Pierce. To compare the monoclonal and polyclonal hybridizations, the membranes were stripped after monoclonal hybridization and then reprobed with the polyclonal antibody. PGIS samples were always hybridized first with the PGIS antibody, then stripped one or even two times before staining with the anti-3-nitrotyrosine antibody. Epitope recognition of nitrated PGIS depends on strong denaturing conditions (SDS in the stripping buffer) to achieve a complete unfolding of the protein, which allows tight binding of the anti-3-nitrotyrosine antibody.

### 4.6. Proteolytic Digestion of Nitrated Proteins by Pronase and Direct Detection of 3-Nitrotyrosine

Pronase digestion was performed as previously described for P450_BM-3_ and P450_CAM_[33,34]. Briefly, nitrated PGIS (3.3 μM) in 0.1 M potassium phosphate buffer pH 7.4 was prepared for the total protein hydrolysis by pronase: 1 mM CaCl_2_ for the stabilization of the proteases was added together with 5% *v*/*v* acetonitrile for protein denaturation and better solubility of the liberated amino acids. These samples were incubated with 2 mg/mL pronase for 24 h at 37 °C, followed by another addition of 2 mg/mL pronase. This last step was repeated once after 48 h. The digestion of PGIS was complete after 3 days whereas for other P450 proteins it was already complete within 2–3 h after addition of pronase. The digested samples were depleted from residual proteins by size exclusion centrifugation with a 10 kDa cut-off (Microcon, Millipore Corporation, Bedford, OH, USA) and the eluates were kept at 4 °C and measured within the next 48 h. 3 and 30 kDa devices were also tested. Several experiments have been conducted to ensure that hydrolysis was complete under these conditions within the indicated time scale. An aliquot (100 μL) of each sample was analyzed by HPLC as described previously [26]. Examples of HPLC chromatograms are shown for P450_CAM_ and BSA in the online supplement (Figures S13 and S14).

### 4.7. Stability of the Nitrated Peptide from PGIS and Specificity of Cleavage by Thermolysine

Two peptides with the sequence of bovine PGIS and inserted 3-nitrotyrosine have been purchased from Jerini (Berlin, Germany). The first one contained 4 amino acids NH_2_-LKNY(NO_2_)-OH (*MW* = 581.3 g/mol) and the second one contained 16 amino acids NH_2_-KDGKRLKNY(NO_2_)NMPWGAG-OH (*MW* = 1879.3 g/mol). To test the stability of the short peptide and the selectivity of cleavage in the long peptide both were subjected to thermolysine digestion. 10 μM of peptide in 50 mM Tris pH 8.0, 4 mM CaCl_2_ and 10% *v*/*v* acetonitrile were incubated for 24 h at 50 °C together with thermolysine (25 μg/mL). After this time period another 125 μg/mL thermolysine were added and incubation was continued for 60 h at 37 °C. The mass spectrometry analysis was performed by Dr. Andrea Kiehne from the application department of Bruker Daltonics GmbH (Bremen, Germany). The samples were diluted in 0.1% formic acid in water yielding a final peptide concentration of 500 fmol/μL. 1 μL of these samples was injected into a NanoLC-MS system esquire 3000 plus from Bruker Daltonics (Bremen, Germany). The conditions were as follows: A C_18_ PepMap (15 cm × 75 μm inner diameter) column and a mobile phase gradient (A: 0.1% formic acid in water, B: 0.1% formic acid in 90% acetonitrile, 0–15 min 2% B isocratic, 15–25 min 2%–90% B, 25–35 min 90% B isocratic, 35–40 min 90%–2% B and 40–70 min 2% B isocratic). The flow rate was 200 nL/min and optical detection at 214 nm was used. The parameters for mass spectrometry analysis were determined by a direct injection experiment. Peptides were identified by full scan MS as well as MS/MS with ion capture at 291.6 *m*/*z*.

### 4.8. Alcohol Dehydrogenase Activity

The activity of 26 nM ADH with 300 μM NAD^+^ for the conversion of 172 mM ethanol to acetaldehyde in 0.1 M potassium phosphate buffer pH 7.6 was followed by spectroscopy at 37 °C on an Aminco DW-2 dual beam spectrophotometer equipped with a magnetic stirrer and connected to a computer. We measured the kinetics of NADH formation at 340 nm and the basic specific activity was calculated from the slope of the linear part of the curve (first 25 s) to be 245 ± 20 U/mg. In a next step the amount of peroxynitrite necessary for a complete inhibition of the enzyme was estimated. Therefore the ethanol and NAD^+^ were added after complete reaction of the ADH with peroxynitrite (5 min) and the activity was measured. We found that 20 μM peroxynitrite quantitatively inhibited ADH. In the following step we estimated the IC_50_-values of several peroxynitrite-scavengers in this system by addition of different concentrations of scavenger to the ADH, before the addition of peroxynitrite. The IC_50_-value of the scavenger corresponds to the concentration at which 50% of the ADH activity is preserved.

### 4.9. Nitration of Phenol

Five millimolars of phenol and 800 μM peroxynitrite were incubated in 0.5 M potassium phosphate buffer pH 7 at 25 °C. The formation of 2- and 4-nitrophenol was followed by spectrophotometry at 405 nm (after addition of 2% *v*/*v* saturated NaOH) or by separation of 2- (*t*_R_ = 28 min) and 4-nitrophenol (*t*_R_ = 16 min) by HPLC (system II, 1 mL/min, 30% acetonitrile and 75% 0.1 M citrate buffer pH 2, Bischoff C_18_-Nucleosil-100-5 250 × 4.6, detection at 280 nm). For the IC_50_-values, the scavengers were added before addition of peroxynitrite by vortex-mixing and IC_50_-values correspond to the concentrations of scavengers, at which the 405 nm absorbance or nitrophenol peak-area were 50% of that measured in the absence of scavengers. 5 mM phenol was incubated either with 655 μM peroxynitrite in 0.2 M potassium phosphate buffer pH 6 (1 min) or with 400 μM peroxynitrite at pH 9 (10 min) and 37 °C. At pH 6 the formation of 2- (*t*_R_ = 15.6 min) and 4-nitrophenol (*t*_R_ = 12.8 min) was monitored by HPLC (system I, 1 mL/min, 10% acetonitrile and 90% 0.05 M citrate buffer pH 2, Macherey-Nagel C_4_-Nucleosil-300-5 250 × 4.6, detection 280 nm). At pH 9 the formation of 4-nitrosophenol (*t*_R_ = 11 min) was either followed vy spectrophotometry at 395 nm or by HPLC (system I, 1 mL/min, 10% acetonitrile and 90% 0.05 M citrate buffer pH 2, Macherey-Nagel C_18_-Nucleosil-100-5 250 × 4.6, detection 300 nm). The IC_50_-values were estimated as described above.

### 4.10. Metal-Catalyzed Nitration of Phenol

The method used was similar to the one used in the phenol system at pH 6, but with 5 μM microperoxidase (MP-11) (a porphyrin with 11 amino acids originating from the degradation of cytochrome c). MP-11 like other heme containing enzymes and iron-porphyrins leads to a strong increase in nitration of phenol. No IC_50_-values were estimated, but the effect of 100 μM scavenger on nitration was monitored using HPLC.

### 4.11. Kinetics of Peroxynitrite-Decomposition

Decay of peroxynitrite was followed by UV/Vis spectroscopy by the difference in absorbance at 302 nm and the reference wavelength at 400 nm. The samples were measured in a special cuvette which was equipped with a magnetic stirrer. Because of inadequate mixing during the first 2–3 s the decomposition curves were only compared qualitatively and no kinetic constants could be obtained from these measurements. 400–800 μM peroxynitrite was injected into a stirred pH 7, 8 or 9 buffer solution which contained the scavenger at different concentrations. The velocity of decay was then compared with the one in controls.

## 5. Conclusions

The results of the present study show that metal catalysis adds efficacy and selectivity to peroxynitrite-mediated nitration reactions. Partially purified PGIS showed complete nitration at a peroxynitrite concentration of 10 μM (3-fold molar excess). This was surprising since the nitrotyrosine formation took place under reducing (antioxidant) conditions mimicked by the microsomes’ sulfhydryl environment thereby underlining the high velocity and specificity of this nitration reaction. We also identified carbon dioxide mediated catalysis of PGIS nitration as an alternative or synergistic pathway of PGIS nitration besides the metal-catalyzed nitration process under physiological conditions. Although the carbon dioxide mediated nitration of PGIS was also inhibited by an active site inhibitor of PGIS, U-51605, it remains to be established whether carbon dioxide-catalyzed nitration targets the same tyrosine residue as the metal-catalyzed process since previous reports did not observe an effect of carbon dioxide on inactivation of PGIS by peroxynitrite [29]. In contrast, we can exclude a major contribution of freely diffusing •NO_2_-radicals to the nitration of PGIS since peroxidase/nitrite/hydrogen peroxide systems were rather inefficient in conferring this nitration process. A valid model for PGIS with respect to nitration is the bacterial monooxygenase P450_BM-3_. Activated thio-compounds such as 2,6-dithiopurine and -pyrimidine proved to be highly efficient scavengers of the various forms of peroxynitrite: the anion (ONOO^–^), the protonated form (ONOOH) as well as derived free radicals (HO• and •NO_2_). All other compounds had more or less pronounced preferences for one of the peroxynitrite species: thiol- and seleno-compounds as well as thioethers reacted most efficiently with the anion whereas they only partially inhibited free radical-mediated reactions such as phenol nitration and hydroxylation. Typical one-electron reductants such as ascorbate and uric acid efficiently suppressed nitration and hydroxylation of phenol. However, it was surprising that structurally-related purines such as xanthine and caffeine did not mimic the effects of uric acid.

## Supplementary Information



## Figures and Tables

**Table 1:** Half-maximal inhibition concentrations (IC_50_-values) of various compounds for the nitration (pH 6) and nitrosation (pH 9) of phenol by peroxynitrite *.

Scavenger	IC_50_ (μM)	Scavenger	IC_50_ (μM)
Glutathione	181 ± 20/380 ± 57	Methionine	450 ± 34/690 ± 75
Ascorbate	88 ± 18/133 ± 18	Uric acid	40 ± 10/57 ± 8
2,6-Dithiopurine	35 ± 13/36 ± 14	2,6-Dithiopyrimidine	32 ± 4/43.5 ± 9.5
Ebselen	128 ± 5/190 ± 11	1,3-Dimethyluric acid	36.5 ± 1.5/25 ± 12
Se-methionine	250 ± 13/170 ± 28	Xanthine	>1 mM/>1 mM
Cysteine	64 ± 14/425 ± 125	3,9-Dimethyluric acid	141 ± 7/n.d.
Alloxan	>1 mM/n.d.	3,7-Dimethyluric acid	19 ± 3/n.d.
2-Thiobarbituric acid	37 ± 6/26 ± 4	Allopurinol	>1 mM/>1 mM
Caffeine	>1 mM/n.d.	Allantoin	>1 mM/n.d.

* First value for nitration (pH 6), second value for nitrosation (pH 9). 5 mM phenol were reacted with 655 μM peroxynitrite at pH 6 and 400 μM peroxynitrite at pH 9. n.d. means not determined.

**Table 2:** Half-maximal inhibition concentrations (IC_50_-values) of various compounds for the nitration of bovine serum albumin (15 μM) by peroxynitrite (1 mM) at pH 7.

Scavenger	IC_50_ (μM)	Scavenger	IC_50_ (μM)
Glutathione	150 ± 26	Methionine	200 ± 38
Ascorbate	170 ± 17	Uric acid	75 ± 13
2-Thiobarbituric acid	45 ± 12	1,3-Dimethyluric acid	60 ± 14
Ebselen	180 ± 12	3,7-Dimethyluric acid	185 ± 37
Xanthine	>500	3,9-Dimethyluric acid	>200
Allopurinol	>500	-	-

**Table 3:** Half-maximal inhibition concentrations (IC_50_-values) of various compounds for the inactivation of alcohol dehydrogenase (26 nM) by peroxynitrite (20 μM).

Scavenger	IC_50_ (μM)	Scavenger	IC_50_ (μM)
Glutathione	31 ± 2.5	Methionine	185 ± 7
Ascorbate	***	Uric acid	400/26 ± 2 *
2,6-Dithiopurine	40 ± 5 **	2,6-Dithiopyrimidine	45 ± 8 **
Ebselen	***	3,7-Dimethyluric acid	400/42 ± 1.5 *
Se-methionine	15 ± 1	Tryptophan	400/35 ± 3 *
Cysteine	18 ± 3	Tyrosine	420 ± 30

* highest concentration of scavenger/percentage of preserved ADH-activity at this concentration;

** scavenger itself reduced ADH-activity by 20% when used at 50 μM;

*** strong inhibition of ADH by ebselen and probably a product from the peroxynitrite-ascorbate reaction.

## Abbreviations

PGIS: prostacyclin synthase
MnSOD: manganese superoxide dismutase
NOS: nitric oxide synthase
P450_BM-3_: cytochrome P450 bacterial monooxygenase-3
P450_CAM_: cytochrome P450 camphor-5-monooxygenase
Sin-1: 3-morpholino sydnonimine
BSA: bovine serum albumin
ADH: alcohol dehydrogenase
MP-11: microperoxidase
