# Supplementary material for: Protein Tyrosine Nitration and Thiol Oxidation by Peroxynitrite—Strategies to Prevent These Oxidative Modifications
Source: Int J Mol Sci. 2013 Apr 8;14(4):7542–70. doi: 10.3390/ijms14047542 (PMC3645702; doi:10.3390/ijms14047542)
Supplement: Supplementary file 1 [file ijms-14-07542-s001.pdf]

Supplementary Information

1. Extended Results

1.1. P450$_{BM-3}$ as a Model of PGIS Nitration by Peroxynitrite

P450 bacterial monooxygenase-3 (P450$_{BM-3}$) is a fused protein with the reductase domain attached to the oxygenase domain which obviously undergoe cleavage in the presence of proteases (Figure S5, upper left). In analogy to Sin-1 stained bands could be observed when the xanthine oxidase/spermine NONOate system was used as a source for peroxynitrite *in situ* formation (Figure S5, lane 3, 5, 8 and 10). In the presence of xanthine oxidase a splitting of the fused P450$_{BM-3}$ F87Y mutant into the reductase domain (not stained) and the oxygenase domain (stained at ~50 kD) could be observed (Figure S5, lane 3).

It is known that xanthine oxidase preparations contain proteases [104] and according to this fact the splitting could be prevented by addition of protease inhibitors (Figure S5, lane 6–10). NO from spermine NONOate alone only caused a weak background staining (lane 2 and 7) and $O_2\cdot^-$ from xanthine oxidase alone at least with the monoclonal antibody caused no staining at all (Figure S5, lane 1 and 6). Cu,Zn-SOD could efficiently block the nitration by peroxynitrite generation from $O_2^-\cdot$ and NO (Figure S5, lane 4 and 9). Qualitatively similar results could be obtained after stripping of the membrane and incubation with polyclonal 3-nitrotyrosine antibody but the intensity of the bands varied less (Figure S5, lower left). The polyclonal antibody even stained bands in the untreated controls (Figure S5, lane 1 and 6) and also the spermine NONOate treated samples (lane 2 and 7) and SOD treated samples (lane 4 and 9) were detected as 3-nitrotyrosine-positive. The splitting of P450$_{BM-3}$ in the presence of xanthine oxidase and absence of protease inhibitors could also be observed on the Ponceau S stained membrane (Figure S5, right). Similar results could be obtained when P450$_{BM-3}$ wild type was treated with Sin-1 or xanthine oxidase/spermine NONOate but the staining was less pronounced as compared to the F87Y mutant (Figure S6).

1.2. Stability of Nitrated PGIS Peptide and Usefulness as a Biomarker for Peroxynitrite in Vivo

In a recent publication we have identified Y_{430} in PGIS as the primary target of tyrosine nitration by peroxynitrite [41]. Since 3-nitrotyrosine formation in PGIS was neither observed in response to hydrogen peroxide/nitrite nor in response to NO formation alone, nitrated PGIS can be predicted to be a suitable biomarker of peroxynitrite formation *in vivo*. This kind of biomarker is of special interest since peroxynitrite formation was proposed to be associated with a huge number of cardiovascular, neurodegenerative and inflammatory diseases which are related to oxidative stress [10,18,19,20,21]. Accordingly, nitrated PGIS has been observed in several of these pathophysiological conditions such as atherosclerosis, diabetes mellitus, ischemia reperfusion, nitrate tolerance as well as cytokine-triggered inflammation (septic shock) [105–109]. We here demonstrate on a chemical basis that digest of a tyrosine-nitrated peptide with the sequence of PGIS (H-KDGKRLKNY$_{430}$(NO_2)NMPWGAG-OH) by thermolysin results in the formation of the stable peptide H-LKNY$_{430}$(NO_2)-OH which probably is suitable as a biomarker of peroxynitrite-derived nitration *in vivo*. Figure S4 shows the LC-MS chromatograms of H-LKNY$_{430}$(NO_2)-OH in the presence or absence of thermolysin clearly indicating that this peptide is not further digested by this protease (Figure S4a,b). H-KDGKRLKNY$_{430}$(NO_2)NMPWGAG-OH only showed the signal of the small nitrated peptide

(m/z = 2916) upon long-term treatment with thermolysin indicating a specific cleavage of the long peptide yielding H-LKNY$_{430}$(NO$_2$)-OH (Figure S4c,d). According to these results one may suggest that thermolysin digest of inflammatory or other oxidative stress subjected tissue results in the formation of the stable biomarker H-LKNY$_{430}$(NO$_2$)-OH indicating nitrated PGIS and accordingly the *in vivo* formation of peroxynitrite.

Figure S1. Detection and quantification of tyrosine nitration in human PGIS. Western blot analysis of microsomal fractions (1 mg/mL total protein) containing 200 µg/mg PGIS which were treated with increasing amounts of authentic peroxynitrite (PN, 0–20 µM) or Sin-1 (0–100 µM). The figure shows the stainings with a polyclonal PGIS antibody (**Panel I**) and a monoclonal 3-nitrotyrosine antibody (**Panel II**). Data are representative for three independent experiments. Adapted from [9].

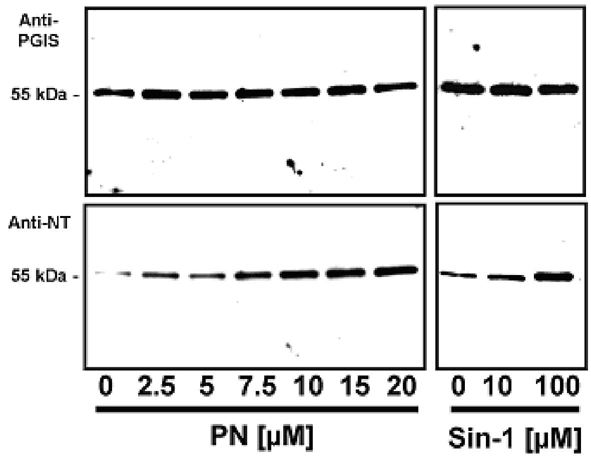

Figure S2. (**Right**) Samples from Western blot analysis shown in Figure V (20 µM Hb + 100 or 2000 µM peroxynitrite) were denaturated in the presence of 10% acetonitrile for 5 min at 95 °C, digested with 0.5 µM trypsin over night and subjected to HPLC analysis and 3-nitrotyrosine containing peptides were detected at 365 nm. Essentially 3 peaks could be identified which increased in dependence of peroxynitrite concentration. (**Left**) The relative area of these 3 peaks was determined in dependence of peroxynitrite concentration.

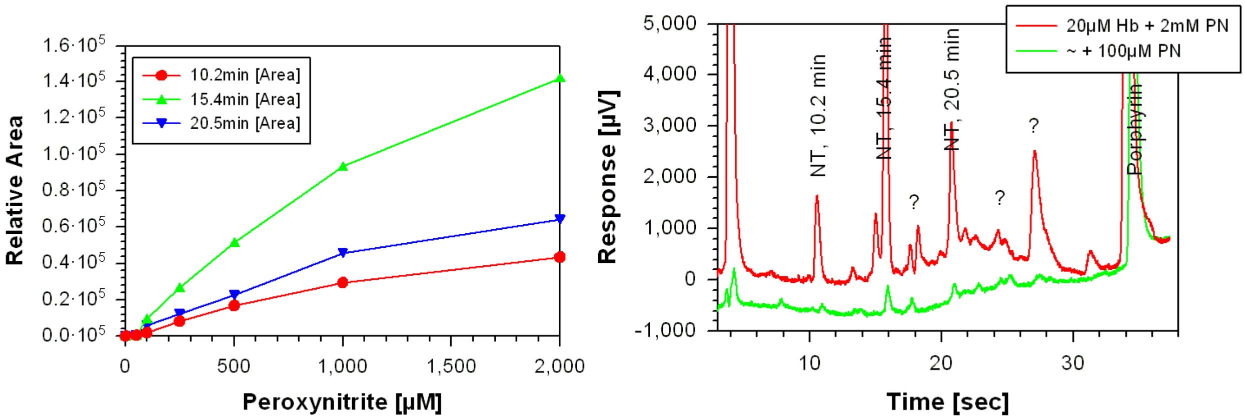

Figure S3. Ponceau S staining corresponding to the Western blot shown in Figure 2A in the manuscript. Lane 1 contained the molecular weight markers, lane 2–5 BSA (5 μM, band between 50 and 75 kDa) and lane 6–9 BSA (5 μM) together with bovine aortic microsomes (1 mg/mL total protein, additional band between 37 and 50 kDa).

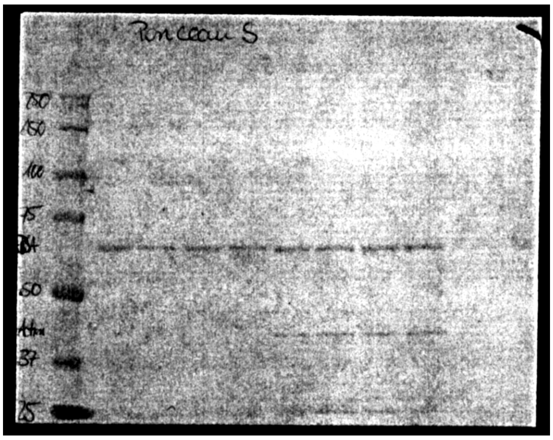

Figure S4. Stability of NH_2-LKNY(NO_2)-OH upon extended incubation with thermolysin and selectivity of cleavage of NH_2-KDGKRLKNY(NO_2)NMPWGAG-OH by thermolysin. The chromatograms show the full scan MS signal (EIC 291.6 *m*/*z*) of NH_2-LKNY(NO_2)-OH (500 fmol) in the absence (**A**) or presence (**B**) of thermolysin. This peptide is also formed from NH_2-KDGKRLKNY(NO_2)NMPWGAG-OH (500 fmol) in the presence (**D**) but not in the absence (**C**) of thermolysin. The insert in (**A**) shows the background subtracted MS spectrum of NH_2-LKNY(NO_2)-OH recorded using the following conditions: capillary exit 125 V, accumulation time 866 μs, ion polarity positive, scan begin 50 *m*/*z*, skim 140 V, averages 8 spectra, scan end 2000 *m*/*z* and trap drive 36.5. Data are representative of at least three independent experiments.

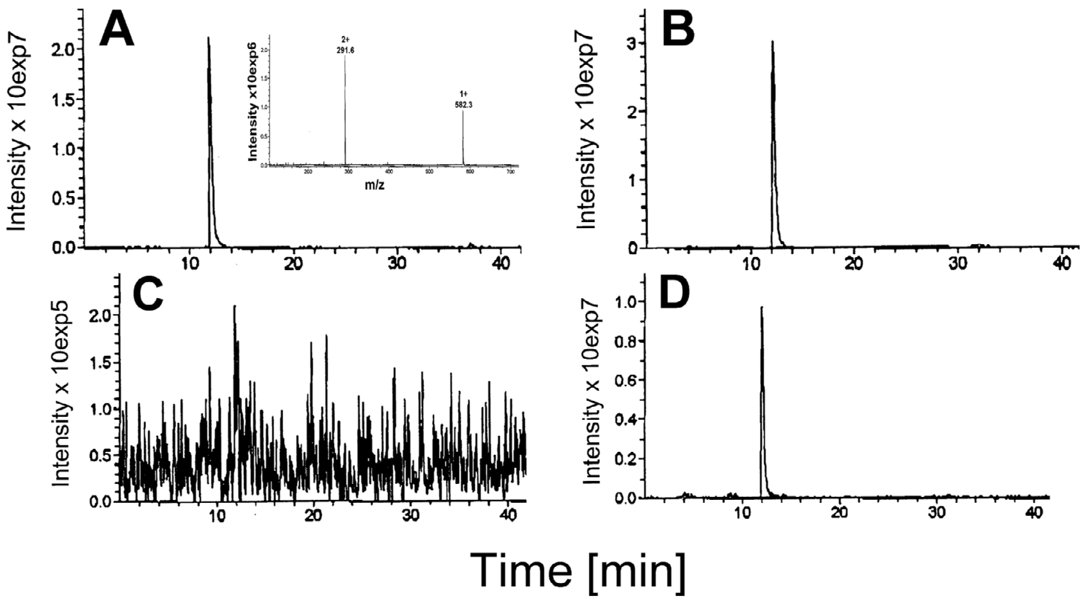

Figure S5. Tyrosine nitration in P450$_{BM-3}$ F87Y variant by *in situ* generated peroxynitrite. Detection of 3-nitrotyrosine in P450$_{BM-3}$ F87Y variant by Western blot analysis using a monoclonal (**upper left**) or polyclonal (**lower left**) 3-nitrotyrosine antibody. P450$_{BM-3}$ F87Y variant (2 µM) was incubated with xanthine oxidase (XO) and/or spermine NONOate in the absence or presence of Cu,Zn-SOD. Sin-1 was used as a positive control of *in situ* peroxynitrite formation. Samples on the left-handed side of the blot (lanes 1–5) were incubated in the absence of protease inhibitors whereas samples on the right-handed side of the blot (lanes 6–10) were incubated in the presence of protease inhibitors. Right: Corresponding Ponceau S stainings indicating proteolytic cleavage in lanes 1, 3 and 4. Data are representative for 3-4 independent experiments.

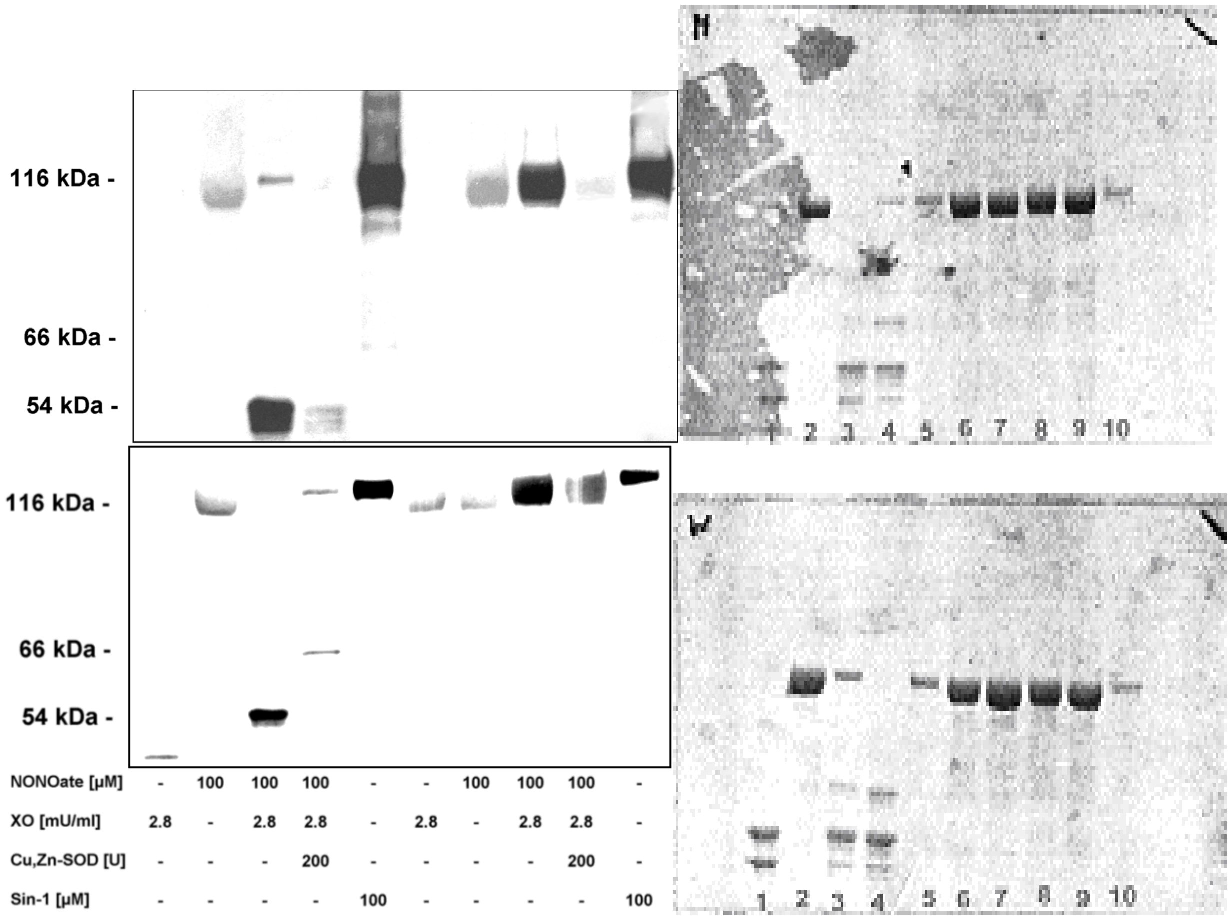

Figure S6. Tyrosine nitration in $P450_{BM\text{-}3}$ wild type by *in situ* generated peroxynitrite. Detection of 3-nitrotyrosine in $P450_{BM\text{-}3}$ wt by Western blot analysis using a monoclonal 3-nitrotyrosine antibody. All other conditions and observations as described in legend to Figure S6.

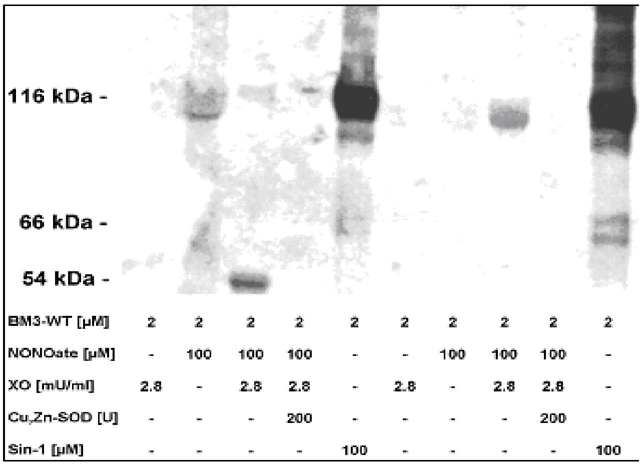

Figure S7. Protein tyrosine nitration was assessed by Western blot analysis using a monoclonal (upper panels) or polyclonal (lower panels) 3-nitrotyrosine antibody. Proteins (5 µM) were incubated with bolus authentic peroxynitrite (500 µM) for 5 min at 37 °C or with *in situ* generated peroxynitrite from Sin-1 (250 or 500 µM) for 90 min at 37 °C in K-phosphate buffer pH 7.4. It is obvious that especially the heme-thiolate proteins $P450_{CAM}$ and $P450_{BM\text{-}3}$ are most selectively nitrated by low steady-state peroxynitrite concentrations from Sin-1. For these proteins we have previously demonstrated metal-catalyzed nitration. The abbreviations are: CAM, $P450_{CAM}$; BM3, $P450_{BM\text{-}3}$; Hb, hemoglobin; Cyt, cytochrome c; ADH, alcohol dehydrogenase; HRP, horseradish peroxidase; Cat, catalase; BSA, bovine serum albumin.

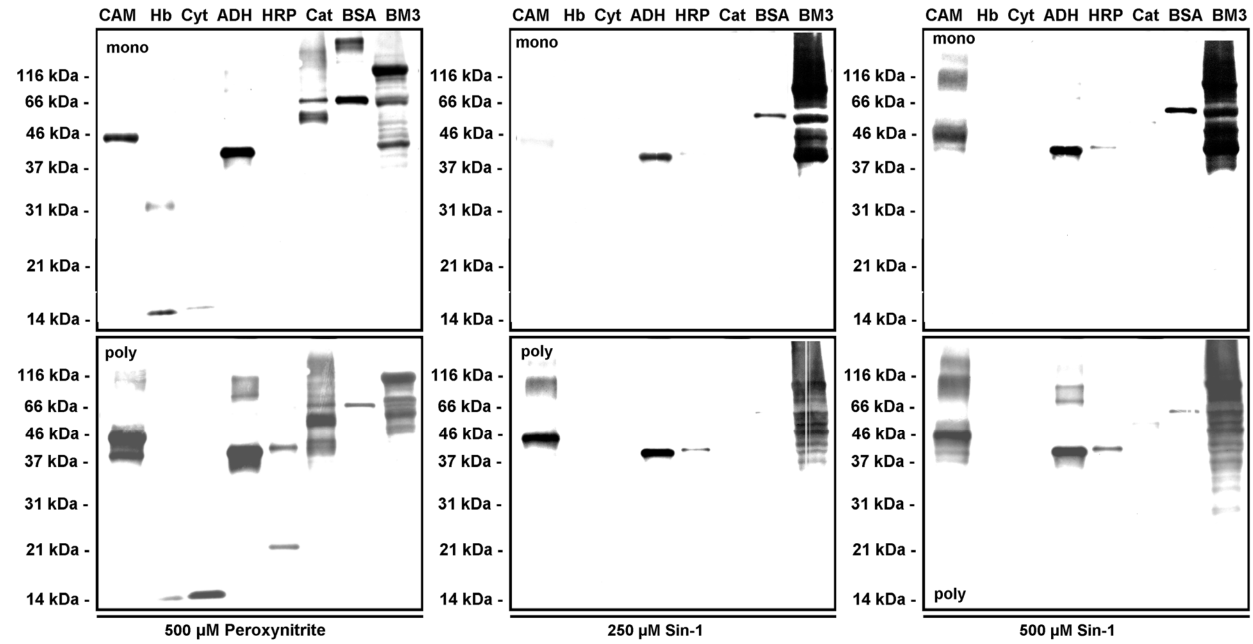

Figure S8. One set of experiments for determination of IC_{50}-values of various compounds for peroxynitrite-mediated nitration. There are clearly two types of scavengers. The first one leads to more than 90% inhibition of phenol nitration at a concentration of 100 µM and shows exponential concentration-inhibition relationship (e.g., uric acid, 2,6-dithiopurine), the other one requires rather high concentrations to reach full protection and rather shows a linear concentration-inhibition relationship (e.g., ebselen, GSH).

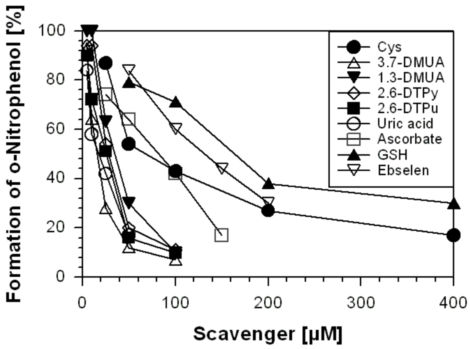

Figure S9. The inhibitory effect of different compounds was tested on peroxynitrite (655 µM) dependent nitration and hydroxylation of phenol (5 mM) at pH 6. The most efficient inhibitors of one-electron oxidations were uric acid, 2,6-dithiopyrimidine and 3,7-dimethyluric acid. Abbreviations are: 2-MBS, 2-mercaptobenzselenazol; 2-MBT, 2-mercaptobenzthiazol; GSH, glutathione; L-Cys, L-cysteine; UA, uric acid; 2-TBA, 2-thiobarbituric acid; L-Met, L-methionine; 2,6-DTPy, 2,6-dithiopyrimidine; 3,7-DMUA, 3,7-dimethyluric acid. * indicates that compounds interfered with peaks of hydroxyphenols and *p*-benzoquinone.

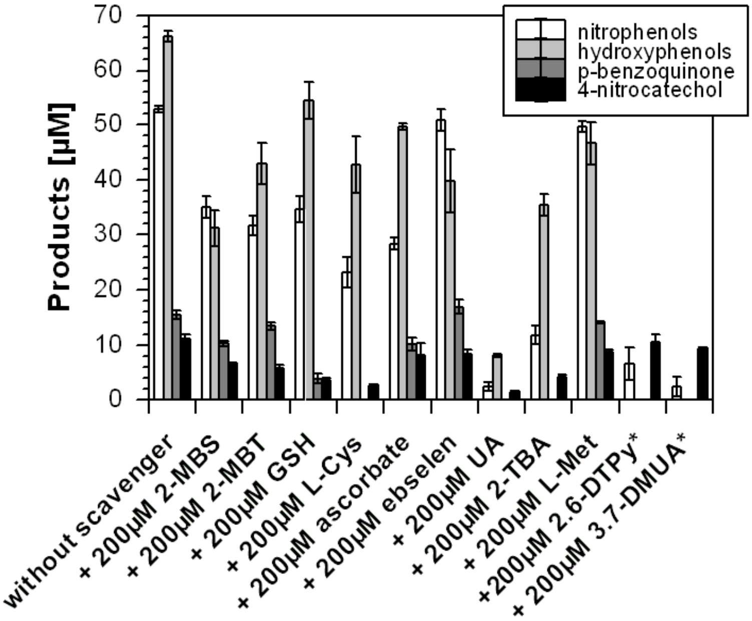

Figure S10. The inhibitory effect of different compounds was tested on peroxynitrite (655 µM) dependent nitration of phenol (5 mM) at pH 6 in the presence of microperoxidase (MP-11), an iron-porphyrin with 11 amino acids. MP-11 increased the nitration of phenol almost 5-fold. The most efficient inhibitors of metal-catalyzed nitration were uric acid, 2,6-DTPu and -DTPy as well as 1,3- and 3,7-DMUA. Abbreviations are: Se-met, seleno-methionine; BPR6, experimental vitamin D derivative; 2,6-DTPu, 2,6-dithiopurine; 1,3-DMUA, 1,3-dimethyluric acid; for others see legend to Figure S10.

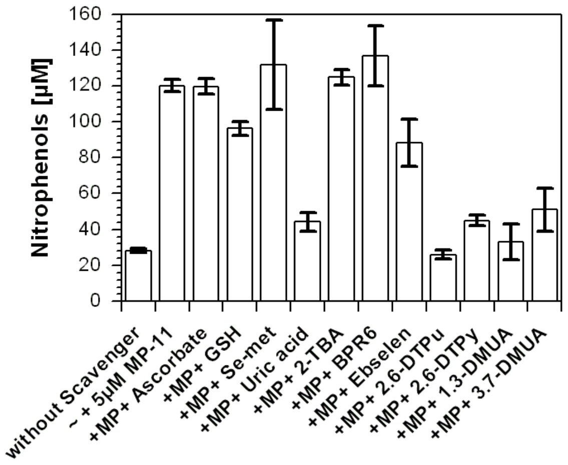

Figure S11. (**Left**) The inhibitory effect of peroxynitrite on alcohol dehydrogenase (ADH, 26 nM) activity. The IC_{50}-value was between 1–2 µM. Since ADH activity requires reduced thiol groups at the active site this enzymatic model was used to study peroxynitrite-mediated sulfoxidation and effects of scavengers of the peroxynitrite anion; (**Right**) The inhibitory effect of ebselen on alcohol dehydrogenase (ADH, 26 nM) activity. The IC_{50}-value was between 50–70 nM. Ebselen is highly reactive towards thiols and forms seleno-thiol-adducts. PON means peroxynitrite.

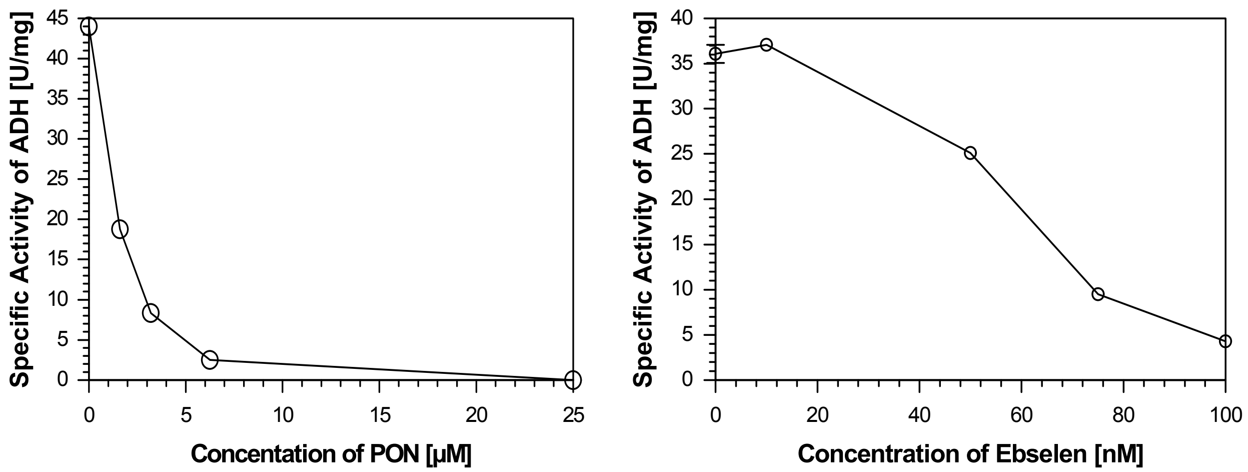

Figure S12. One set of experiments for determination of IC_{50}-values of various compounds for peroxynitrite-mediated inactivation of ADH. 400 µM GSH were roughly half-maximal protective, whereas ebselen directly inhibited the enzyme and ascorbate probably by formation of ascorbyl radicals also lead to inactivation. ADH activity was measured by NADH absorbance at 340 nm upon conversion of ethanol to acetaldehyde.

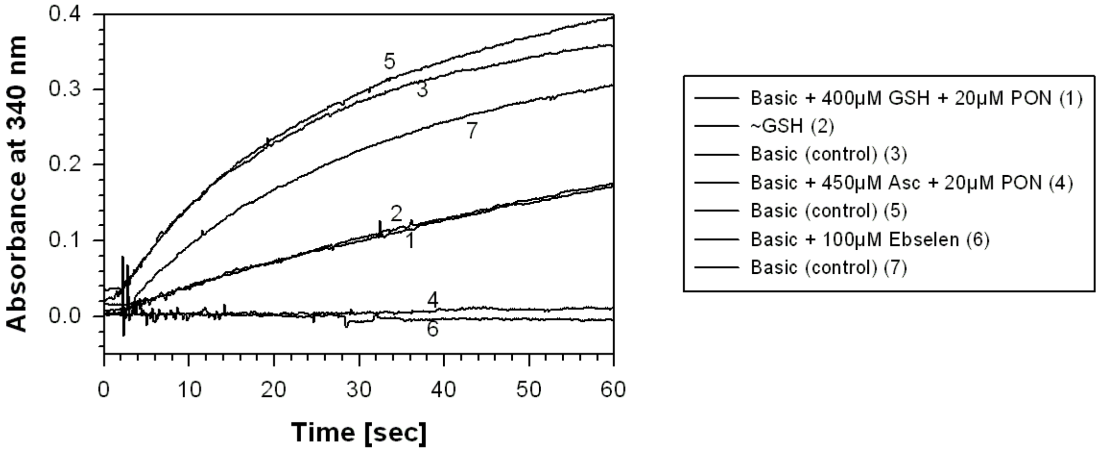

Figure S13. Signal/noise ratio in HPLC chromatograms with optical detection of free 3-nitrotyrosine at 365 nm (**Left**) and its anion at 428 nm (**Right**). It is obvious that interference with other peaks is decreased and accordingly sensitivity is increased with detection at 428 nm. Conditions: 2.5 µM $P450_{CAM}$ were nitrated with peroxynitrite (10–100 µM), digested with pronase and subjected to HPLC analysis using a C_{18} Nucleosil (125 × 4.6) 100-3 column, a flow rate of 0.8 mL/min and a gradient (0–8 min 95 *vv*% 50 mM potassium phosphate buffer pH 6.0 and 5 *vv*% acetonitrile) with postcolumn alkalinization by 0.25 mL/min 100 mM Tris pH 10.5.

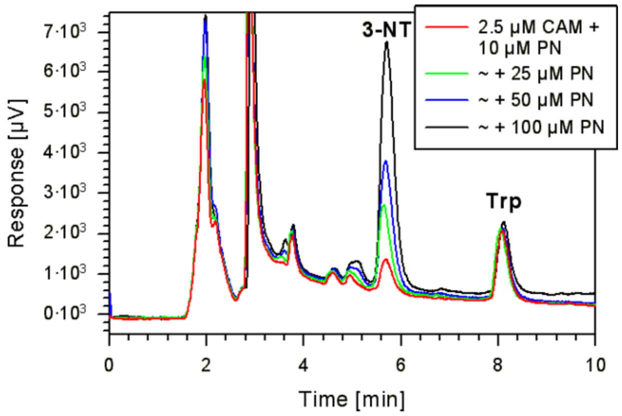

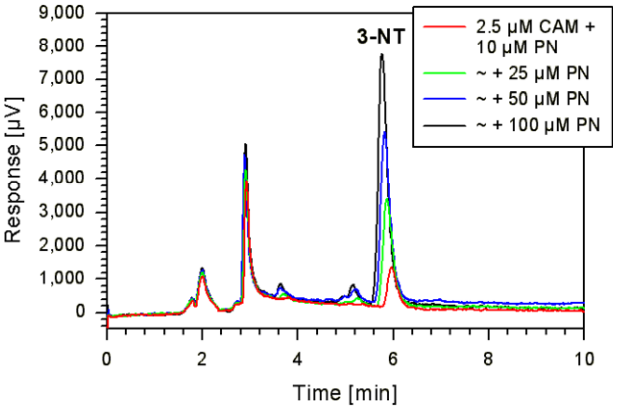

Figure S14. (**Left**) Purification of pronase-digested samples increases sensitivity for free 3-nitrotyrosine. The red trace shows the chromatogram of authentic 3-nitrotyrosine (1 µM). The green trace shows the peak broadening in the presence of a pronase digest of BSA (5 µM) which was improved (blue trace) by purification using size exclusion centrifugation through a 10 kDa Microcon filter device from Millipore (Bedford, OH, USA) and normalized upon size exclusion centrifugation through a 3 kDa Microcon filter device. Digest conditions: 2 mg/mL pronase, 1 mM $CaCl_2$ and 5% acetonitrile for 4 h at 37 °C; (**Right**) The kinetics for pronase digest of nitrated (1 mM peroxynitrite) BSA and HSA are also shown.

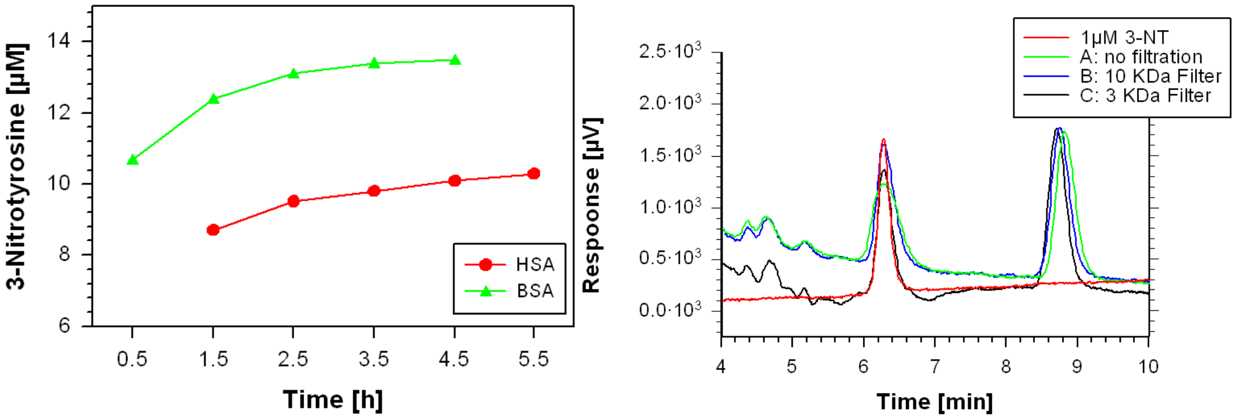

© 2013 by the authors; licensee MDPI, Basel, Switzerland. This article is an open access article distributed under the terms and conditions of the Creative Commons Attribution license (http://creativecommons.org/licenses/by/3.0/).
